# A Prospective Study of Different Types of Dietary Fiber and Risk of Cardiovascular Disease: Tehran Lipid and Glucose Study

**DOI:** 10.3390/nu8110686

**Published:** 2016-11-07

**Authors:** Parvin Mirmiran, Zahra Bahadoran, Sajad Khalili Moghadam, Azita Zadeh Vakili, Fereidoun Azizi

**Affiliations:** 1 Nutrition and Endocrine Research Center, Student Research Committee, Research Institute for Endocrine Sciences, Shahid Beheshti University of Medical Sciences, No. 24, Shahid-Erabi St., Yeman St., Velenjak, Tehran 19395-4763, Iran; mirmiran@endocrine.ac.ir (P.M.); sajadkhalili69@gmail.com (S.K.M.); 2 Cellular and Molecular Endocrine Research Center, Obesity Research Center, Research Institute for Endocrine Sciences, Shahid Beheshti University of Medical Sciences, No. 24, Shahid-Erabi St., Yeman St., Velenjak, Tehran 19395-4763, Iran; azitavakili@endocrine.ac.ir; 3 Endocrine Research Center, Research Institute for Endocrine Sciences, Shahid Beheshti University of Medical Sciences, No. 24, Shahid-Erabi St., Yeman St., Velenjak, Tehran 19395-4763, Iran

**Keywords:** coronary heart disease, dietary fiber, soluble fiber, insoluble fiber

## Abstract

Background and aim: This study was designed to examine the hypothesis that dietary of intake different types of fiber could modify the risk of cardiovascular disease (CVD) in a large prospective cohort among Iranian adults. Methods: In 2006–2008, we used a validated food frequency questionnaire to assess dietary fiber intake among 2295 health professionals with no previous history of heart disease. Subjects were subsequently followed until 2012 for incidence of CVD events. Multivariate Cox proportional hazard regression models, adjusted for potential confounders were used to estimate the risk of CVD across tertiles of total dietary fiber and different types of fiber. Linear regression models were also used to indicate the association of dietary fiber intakes with changes of cardiovascular risk factors during the follow-up. Results: Mean age of participants (42.8% men) was 38.2 ± 13.4, at baseline. Mean (SD) dietary intake of total fiber was 23.4 (8.9) g/day. After adjustment for cardiovascular risk score and dietary confounders, a significant inverse association was observed between intakes of total, soluble and insoluble dietary fiber and CVD risk, in the highest compared to the lowest tertiles (HR = 0.39, 95% CI = 0.18–0.83, HR = 0.19, 95% CI = 0.09–0.41, and HR = 0.31, 95% CI = 0.14–0.69, respectively). Inverse relations were observed between risk of CVD and dietary fiber from legumes, fruits and vegetables; however, dietary fiber intake from grain and nut sources was not related to risk of CVD. Conclusion: Our findings confirmed that higher intakes of dietary fiber from different sources is associated with CVD events and modify its major risk-related factors.

## 1. Introduction

Dietary fiber, by its impact on the glycemic response and other aspects of metabolism, may also have important effects on cardiometabolic pathways [1]. An increasing number of studies have reported a reduced risk of cardiovascular disease (CVD) following regular diets high in fiber. Based on findings from epidemiologic studies regarding the protective effects of fiber intakes, the Dietary Reference Intakes (DRI) recommended consumption of dietary fiber is 14 g/1000 kcal, or 25 and 38 g/day for adult women and men, respectively [2]. Consuming a diet rich in high-fiber foods is also a critical component of the American Heart Association’s strategy and other dietary recommendations for cardiovascular disease risk reduction in the general population [3,4].

It has been proposed that dietary fiber could modify underlying CVD risk factors including lipid and lipoprotein metabolism, insulin homeostasis, inflammatory markers and coagulation, and also improve insulin sensitivity, thereby reducing the risk of CVD mortality [5,6,7]. Although studies showed beneficial effects of soluble, gel-forming fiber on cardiometabolic risk factors, food sources of mainly insoluble fibers, primarily contributed by cereal products, have been the fiber most consistently associated with lower risk of CVD [5]. Findings of some investigations also suggest that the role of dietary fiber is more dependent on its types and sources, rather than the amount of intake [8,9]. Different types or sources of dietary fiber may induce different physiological effects; soluble fiber is responsible for the cholesterol-lowering effect of dietary fiber whereas insoluble fiber interacts with intestinal absorption of foods and contributes to reduction in clotting factors [10,11].

To examine the hypothesis that a greater intake of dietary fiber reduces risk of CVD, and different dietary fiber may lead to different CVD outcomes, we used prospective data from the Tehran Lipid and Glucose Study over a 6-year period to assess the relationship between total dietary fiber, soluble and insoluble fiber, and different fiber sources on the risk of CVD among Iranian adults.

## 2. Methods

### 2.1. Study Population

**Figure 1:**
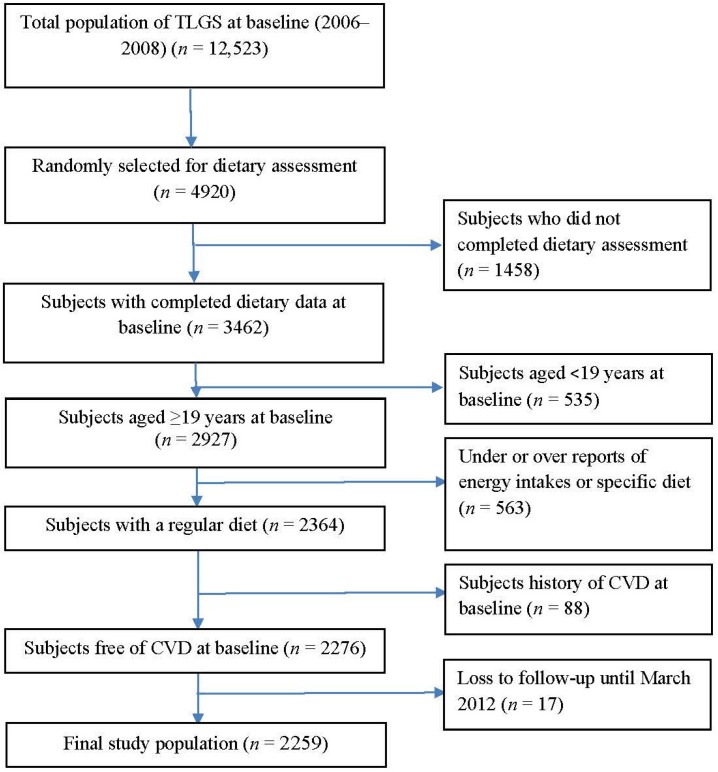
The flowchart of the study population.

This study was conducted within the framework of the Tehran Lipid and Glucose Study (TLGS), an ongoing community-based prospective study being conducted to investigate and prevent non-communicable diseases in a representative sample in the district 13 of Tehran, the capital city of Iran [12]. During the third phase of the TLGS (2006–2008), of a total of 12,523 subjects who completed the examinations, 4920 were randomly selected for completing the dietary assessment based on their age and sex. The randomization was performed because of cost and complexity of dietary data collection in large populations and also the fact that this process is time-consuming. Finally, the dietary data for 3462 subjects who agreed to participate and completed the food frequency questionnaire (FFQ) were available. The characteristics of participants who completed the validated FFQ were similar to those of the total population in the third phase of TLGS [13]. For the purpose of the current study, among subjects aged ≥19 years, we recruited 2927 adult men and women, with complete data (demographics, anthropometrics, biochemicals). Participants were excluded from the final analysis if they had under- or over-reported energy intakes (<800 kcal/day or >4200 kcal/day, respectively), or were on specific diets (*n* = 563). Participants were also excluded if they had history of CVD at baseline examination (*n* = 88). The remaining participants (*n* = 2276) were followed until March 2012, for a mean duration of 4.7 years from the baseline examination. Participants who had left the study (*n* = 17) were also excluded and final analyses was conducted on data of 2259 adults (Figure 1).

Written informed consents were obtained from all participants. The study protocol, based on the ethical guidelines of the 1975 Declaration of Helsinki, was approved (ethics committee number: 57ECRIES94/02/15) by the Ethics Research Council of the Research Institute for Endocrine Sciences, Shahid Beheshti University of Medical Sciences.

### 2.2. Demographic and Anthropometric Measures

Demographics, anthropometrics and biochemical measures were assessed both at baseline (2006–2008) and again at the follow-up examination (2010–2012). Trained interviewers collected information including demographic data, medical history, medication use and smoking habits, using pretested questionnaires. Weight was measured to the nearest 100 g using digital scales, while the subjects were minimally clothed, without shoes. Height was measured to the nearest 0.5 cm, in a standing position without shoes, using a tape meter. Body mass index was calculated as weight (kg) divided by square of the height (m^2^). Waist circumference was measured to the nearest 0.1 cm, midway between the lower border of the ribs and the iliac crest at the widest portion, over light clothing, using a soft measuring tape, without any pressure to the body. For blood pressure (BP) measurements, after a 15-min rest in the sitting position, two measurements of BP were taken on the right arm, during a standardized mercury sphygmomanometer; the mean of the two measurements was considered as the participant’s BP.

### 2.3. Biochemical Measures

Fasting blood samples were taken after 12–14 h from all study participants, both at baseline and follow-up phase. Serum creatinine levels were assayed using kinetic colorimetric Jaffe method. Fasting serum glucose (FSG) was determined by the enzymatic colorimetric method using glucose oxidase. The standard 2 h serum glucose 2-h SG test was performed for all individuals who were not on anti-diabetic drugs. In a subsample of the population (*n* = 904), fasting serum insulin (FSI) was measured, by the electrochemiluminescence immunoasaay (ECLIA), using Roche Diagnostics kits and the Roche/Hitachi Cobas e-411 analyzer (GmbH, Mannheim, Germany). Intra- and inter-assay coefficients of variation for insulin were 1.2% and 3.5%, respectively.

Triglyceride (TG) levels were assessed by enzymatic colorimetric analysis with glycerol phosphate oxidase. High-density lipoprotein cholesterol (HDL-C) was measured after precipitation of the apolipoprotein B containing lipoproteins with phosphotungstic acid. Analyses were performed using Pars Azmoon kits (Pars Azmoon Inc., Tehran, Iran) and a Selectra 2 auto-analyzer (Vital Scientific, Spankeren, The Netherlands). Both inter- and intra-assay coefficients of variation of all assays were <5%.

### 2.4. Dietary Assessment

A 168-item food frequency questionnaire (FFQ) was used at the first examination to assess typical food intakes over the previous year. The validity and reliability of the FFQ had previously been assessed in a random sample, by comparing the data from two FFQs, completed 1 year apart and comparing the data from the FFQs and 12 dietary recalls, respectively; the validity and reliability of the FFQ for total dietary fat were acceptable; correlation coefficients between the FFQ and multiple 24 recalls were 0.59 and 0.38 and those between the two FFQs were 0.43 and 0.42 in male and female subjects, respectively [14]. Study of the reliability, comparative validity and stability of dietary patterns derived from the FFQ also showed that there was a reasonable reliability and validity of the dietary patterns among the population over time [15]. Trained dietitians asked participants to designate their intake frequency for each food item consumed during the past year on a daily, weekly, or monthly basis. Portion sizes of consumed foods reported in household measures were then converted to grams. Energy and nutrient content of foods and beverages were analyzed using the US Department of Agriculture Food Composition Table (FCT) because the Iranian FCT is incomplete, and has limited data on nutrient content of raw foods and beverages [13]. Finally, dietary intakes of participants, including dietary energy and energy density, macronutrients, and micronutrients were determined. In addition to calculating total dietary fiber from all foods, soluble and insoluble dietary fiber intakes were separately calculated. Additionally, we computed dietary fiber from different sources including cereal (traditional breads including barbari, taftoon, sangak, lavash, baguette breads, white rice, and barley), legumes (soya, cowpea, chickpea, broad bean, red bean, white bean, lentil, and split pea), nuts (almond, pistachio, walnut, hazelnut, and peanut), vegetables (green leafy vegetables, roots, starchy vegetables, and other vegetables including cucumber, tomato, scallion, zucchini, eggplant, cauliflower, onion, and garlic) and fruits (cantaloupe, melon watermelon, pear, apricot, cherries, apple, peach, nectarines, figs, grapes, kiwi, grapefruit, tangerine, pomegranate, strawberry, banana, lemons) as well.

### 2.5. Definition of Terms

History of cardiovascular disease was defined as previous ischemic heart disease and/or cerebrovascular accidents. Family history of premature cardiovascular disease reflected any prior diagnosis of cardiovascular disease in first-degree female relatives, aged <65 years, or first-degree male relatives, aged <55 years. Diabetes was defined as fasting serum glucose ≥126, 2 h serum glucose ≥200 or anti-diabetic medications [16]. Hypertension (HTN) was considered as systolic BP ≥140 mmHg or systolic BP ≥90 mmHg or current use of antihypertensive medications [17]. Homeostatic model assessment of insulin resistance was defined as follows: HOMA-IR = fasting insulin (μU/mL) × fasting glucose (mmol/L)/22.5; this index has been developed as a simple, inexpensive, and validated alternative tool for assessment of insulin resistance in epidemiological studies [18,19]. Insulin resistance was defined as HOMA-IR ≥3.2 [20].

### 2.6. Definition of Outcome in Our Study

Details of the collection of cardiovascular outcome data have been described elsewhere [21]. Briefly, each participant was followed up for any medical event annually by phone calls. Information of any medical condition or event was collected by a trained nurse, a trained physician and by utilization of data from medical files. The collected data were evaluated and confirmed by an outcome committee consisting of an internist, an endocrinologist, a cardiologist, an epidemiologist and other experts. CHD were included cases of definite myocardial infarction (MI) (diagnostic ECG and biomarkers), probable MI (positive ECG findings plus cardiac symptoms or signs plus missing biomarkers or positive ECG findings plus equivocal biomarkers), and angiographic proven CHD. Cardiovascular disease was defined as any CHD events, stroke (a new neurological deficit that lasted ≥24 h), or CVD death (definite fatal MI, definite fatal CHD, and definite fatal stroke) [22]. The CVD risk score was calculated according to the sex-specific “general CVD” algorithms were derived that incorporated age, total cholesterol, HDL-C, SBP, treatment for HTN, smoking, and diabetes status [23].

### 2.7. Statistical Methods

Dietary intakes of fiber and other nutrients were adjusted for total energy intake, according to residuals methods [24]. Mean (SD) values and the frequencies (%) of baseline characteristics of the participants, with and without CVD event were compared using independent t test or chi square test, respectively.

Linear associations of baseline intakes of total and different types of dietary fiber with changes of serum lipids, blood pressure and insulin levels during the follow-up period were estimated using linear regression models with adjustment of age, sex, and BMI.

A univariate analysis was performed for potential confounding variables including CVD risk score, dietary intake of total fats (% of energy), sodium (mg/1000 kcal), and vitamin C (mg/1000 kcal); variables with P_E_ < 0.2 in the univariate analyses were selected for the final multivariable models. Adjustment of CVD risk score, as a continuous potential risk factor of CVD, improve the stability of our models in the case of a limited number of events during the study follow-up.

To determine whether the associations of soluble and insoluble fiber intake are independent of each other, we adjusted the models in the presence of each type of the fiber. Similarly, the analyses of fiber from each different source were adjusted for fiber from all of the other sources.

Cox proportional hazard regression was used to assess the hazard ratios (HRs) of dietary fiber intakes for CVD. Time to event was defined by time of censoring or having event, whichever came first. The proportional hazards assumption was tested. We censored participants at the time of other causes of death, leaving the district or being in the study until March 2012 without event. The energy-adjusted amount of dietary fiber and its different types was categorized into tertiles, and the first tertile was given as a reference. Two Cox proportional hazard regression models were defined; model 1 was adjusted for CVD risk score and model 2 was further adjusted for dietary intakes of total fats, sodium and vitamin C. To assess the overall trends of HRs across tertiles of dietary fiber intakes, the median of each tertile was used as a continuous variable in Cox proportional hazard regression models.

All analyses were performed using IBM SPSS for Windows version 19 and STATA version 12 SE (StataCorp LP, College Station, TX, USA), with a two-tailed *p* value < 0.05 being considered significant.

## 3. Results

Mean age of participants (42.8% men) was 38.2 ± 13.4, at baseline. Mean (SD) dietary intake of total fiber was 23.4 (8.9) g/day. Legumes (35.8%), fruits (30.4%), vegetables (27.8%), and grains (24.0%) had greater contributions to total intakes of dietary fiber, respectively. During the average 4.7 ± 1.4 year of follow-up, 57 participants experienced CVD events; of which the more common events were angiographic proven CVD (40.4%), definite MI (24.6%), unstable angina (12.2%) and stroke (8.8%).

The distributions of the major known CVD risk factors and some biochemical values for the participants who had a CVD event and for those who did not are shown in Table 1. Higher prevalence of diabetes (14.3 vs. 3.9, *p* = 0.002) and HTN (43.9 vs. 8.3, *p* = 0.001), as well as higher rate of medications, including lipid-lowering drugs, anti-hypertensive drugs and aspirin, was observed in subjects with CVD events, compared to the rest of the cohort. Compared with non-CVD subjects, CVD patients were more likely to be older, and had higher BMI, waist circumference, blood pressure, serum creatinine, FPG, TG, TG/HDL-C ratio and CVD risk score at baseline (*p* for all < 0.05). Compared to non-CVD subjects, CVD patients had also higher intakes of dietary carbohydrate and lower intake of mono-unsaturated fatty acids and fiber (*p* for all < 0.05).

Results of linear regression showed a significant association between grain fiber intake and baseline CVD risk score (β = 0.05, 95% CI = 0.02–0.08) as well as changes of HOMA-IR (β = 0.04, 95% CI = 0.01–0.08) during the study follow-up. Legume fiber was associated with baseline CVD risk score (β = −0.14, 95% CI = −0.23, −0.04) and changes of HDL-C (β = 0.64, 95% CI = 0.19–1.08). Furthermore, dietary fiber from vegetables (β = −0.24, 95% CI = −0.34, −0.15), nuts (β = −0.23, 95% CI = −0.33, −0.13), fruits (β = −0.18, 95% CI = −0.28, −0.08) was inversely related to CVD risk score at baseline. Higher intake of dietary fiber from vegetables was related to lower levels of TG (β = −7.4, 95% CI = −12.7, −2.1) and TG/HDL-C ratio (β = −0.18, 95% CI = −0.29, −0.03), whereas dietary fiber intake from fruits was inversely related to changes of insulin levels (β = −7.80, 95% CI = −12.8, −2.7) and DBP (β = −0.50, 95% CI = −1.08, −0.03); compared to baseline values, higher intake of fruit-based fiber was also related to higher HDL-C levels (β = 0.54, 95% CI = 0.09–0.98).

There was also a significant negative association between fiber from nuts with body weight changes during the study period (β = −2.01, *p* < 0.05); no significant association was observed between other sources of dietary fiber or total fiber with changes of body weight.

The hazard ratios (95% CIs) of CVD across tertiles of dietary fiber and its categories are shown in Table 2. After adjustment of all potential confounding variables, a lower risk of CVD was observed in the highest compared to the lowest tertile of total dietary fiber intakes (HR = 0.39, 95% CI = 0.18–0.83, *p* for trend = 0.05). Soluble and insoluble dietary fiber was also negatively related to risk of CVD (HR = 0.19, 95% CI = 0.09–0.41, and HR = 0.31, 95% CI = 0.14–0.69) with a significant decreasing trend across increasing intakes (*p* for trend < 0.01).

Table 3 shows HRs (95% CIs) across tertile categories of different dietary sources of fiber. Dietary intake of grain and nut fiber had no significant association with the risk of CVD, whereas legume fiber intake was inversely related to risk of CVD (HR = 0.31, 95% CI = 0.15–0.65, in the third compared to the first tertile, *p* for trend = 0.003); similar associations were also observed for dietary intakes of fiber from fruit and vegetable sources. A combination of dietary fiber intake from vegetables, fruits and legumes also had similar impact on the risk of CVD (HR = 0.65, 95% CI = 0.34–1.24, and HR = 0.46, 95% CI = 0.22–0.96, in the second and third tertiles, respectively, *p* for trend = 0.11).

## 4. Discussion

In this prospective cohort study, conducted on a representative Iranian population, a mean 4.7-year follow-up showed that dietary fiber intake especially from legume, fruit, vegetable and nut sources had protective effect against the development of CVD events. Following adjustment of multiple CVD risk factors and dietary variables, most negative trends in the current study remained statistically significant.

Beneficial effects of vegetable fiber in reduced risk of CVD, in our study, could be related to decreased TG and TG to HDL-C ratio during the study follow-up. Lower risk of CVD across increasing intakes of dietary fiber from fruit sources could also be attributed to its inverse association with insulin levels and DBP as well as positive association with HDL-C. Lack of the beneficial effects of grain fiber on the risk of CVD may be explained by its positive relation with CVD risk score at baseline and its association with increased insulin resistance index during the study follow-up. It should be noted that in our study, high intakes of refined grains such as white rice and white breads had major contributions to dietary intakes of grain fiber, whereas the protective effects of grain fiber, reported in some previous studies, were mainly related to whole grains intake [11]. Aside from chance and biases, another explanation is that other characteristics of refined grains such as high-glycemic index nature, rather than fiber per se, may be responsible for the associations observed.

In a 6-year follow-up study among adult men, higher fiber intake (28.9 vs. 12.4 g/day) was inversely related to risk of total myocardial infarction by 0.41 (RR = 0.59, 95% CI = 0.46–0.76); the inverse association was strongest for fatal myocardial infarction (RR = 0.45, 95% CI = 0.28–0.72) [25]. Each 10-g increase in total dietary fiber corresponded to an RR for total myocardial infarction of 0.81 (95% CI, 0.70–0.93); among the three main food contributors to total fiber intake, cereal fiber had a stronger effect on the reduced risk of total myocardial infarction (RR = 0.71, 95% CI = 0.55–0.91 for each 10-g increase in cereal fiber/day) [25]. In another prospective cohort of elderly men, only cereal fiber consumption was associated with lower incident total stroke and ischemic stroke, whereas neither fruit fiber intake nor vegetable fiber intake were associated with incident CVD; compared to cereal fiber from other sources, fiber from dark breads including wheat, rye, and pumpernickel were associated with a lower risk of CVD (HR = 0.76, 95% CI = 0.64–0.90) [26].

Findings of a recent meta-analysis of 22 prospective cohorts showed that total dietary fiber intake was inversely associated with risk of CVD (Risk ratio = 0.91 per 7 g/day, 95% CI = 0.88 to 0.94) and CVD (Risk ratio = 0.91, 95% CI = 0.87–0.94); insoluble fiber and fiber from cereal and vegetable sources were inversely associated with the incident CVD and CVD, whereas fruit fiber intake was only inversely associated with risk of CVD [27]. No clear differences in the effect of dietary fiber intake from various food groups on CVD mortality have been observed in a cohort of adult men, although every additional 10 g/day intake of dietary total fiber reduced CVD mortality by 17% (95% CI = 2%–30%) and all-cause mortality by 9% (95% CI = 0%–18%) [28] (Table 4). The cardioprotective effect of dietary fiber has been found to be stronger for cereal fiber than for fruit or vegetable fiber [29]. In a cross-sectional study conducted on adult men and women, higher intakes of total fiber and insoluble fiber were inversely related to systolic blood pressure [30].

In our previous study, we showed that higher intake of total dietary fiber reduced the risk of metabolic syndrome by 47% (OR = 0.53, 95% CI = 0.39–0.74, *p* for trend < 0.05); soluble and insoluble fiber intakes were also related to lower risk of metabolic syndrome (OR = 0.60, 95% CI = 0.43–0.84, and OR = 0.51, 95% CI = 0.35–0.72). Similar findings were also observed for dietary fiber from fruits, cereals and legumes sources but not for vegetable and nut fiber [9]. In the National Health and Nutrition Examination Survey, dietary fiber intake was related to a low and intermediate lifetime CVD risk and there was a significant inverse linear association between dietary fiber intake and log-transformed C-reactive protein [31].

Some differences between our findings and those of other cohorts may be related to different dietary habits and dietary patterns; previous studies among Iranians demonstrated some major dietary patterns including traditional dietary pattern with higher load of white rice, traditional breads, vegetables, full-fat dairy products, hydrogenated fats, legumes, dried fruits and nuts; the healthy dietary pattern was related to a higher load of vegetables and fruits, whereas the Western pattern had higher load of fast foods, salty snacks, sweets, mayonnaise, and soft drinks [32,33]. Lack of significant associations between fiber from grains and nuts may be related to types of these food groups consumed among our population. Main grains consumed among the population were refined grains including white rice, and breads with a relatively low-fiber wheat flour; moreover, in our population, nuts were mainly consumed in the form of salty and roasted; these factors may reduce the expected protective effects of fiber from grains and nut sources.

**Figure 2:**
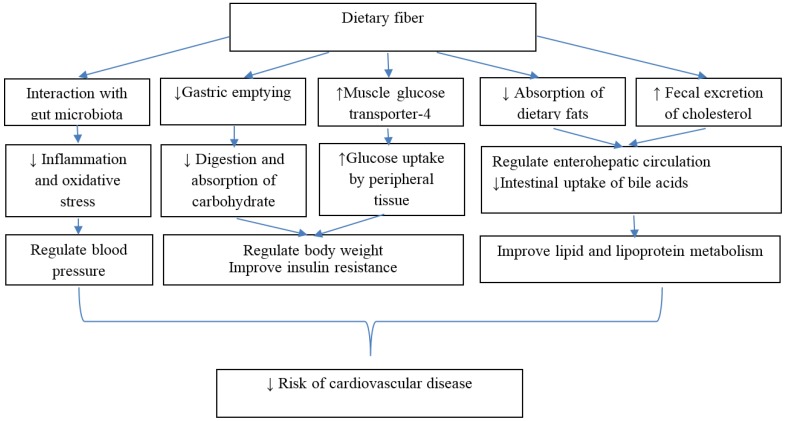
Mechanisms of protective effects of dietary fiber against development of cardiovascular disease. Dietary fiber improved insulin resistance by delaying gastric emptying, reduced absorption and digestion of carbohydrate and increased glucose uptake by peripheral tissue [34]. Dietary fiber also improved lipid and lipoprotein metabolism by decreased absorption of dietary fats, increased fecal excretion of cholesterol and decreased hepatic cholesterol synthesis; dietary fiber especially from cereal sources improved CVD health through multiple mechanisms including lipid reduction, body weight regulation, improved glucose metabolism, blood pressure control, and attenuation of oxidative stress and sub-clinical chronic inflammation [29]. Dietary fiber also modulated gut microbiota and modified cardiometabolic disorders [38,39,40].

Several mechanisms have been proposed to explain beneficial effects of dietary fiber on metabolic pathways; Figure 2 displays some important mechanisms of cardioprotective effects of dietary fiber intakes. Colonic fermentation and subsequent production of short chain fatty acids is another metabolic effect of most types of dietary fiber especially soluble fibers; dietary fiber intake can also regulate gut hormonal responses that may act as satiety factors [34]. The lowering effect of dietary fiber on plasminogen activator inhibitor type 1 and factor VII coagulation activity is also another proposed mechanism for the biological actions of dietary fiber against cardiovascular outcomes [35,36]. Interplay between dietary fiber intakes and the intestinal microbiome has been found to modify the inflammatory responses in the body [37]. Considering the contributory role of gut microbiota in the development of cardiometabolic disorders, such as atherosclerosis, obesity, and type 2 diabetes, and the favorable effects of dietary fiber in modulation of gut microbiota, some cardioprotective properties of dietary fibers may be attributed to this mechanism [38,39,40].

Some strengths and limitations in the current study should be considered. Among the strength, its prospective population-based design, high participation rate and completeness of the follow-up, and use of a validated comprehensive FFQ to assess regular dietary intakes of the participants provided us an opportunity to investigate the associations of total intakes and different types of dietary fiber with 5-year incidence of CVD, relationship that have not been previously examined among Iranian population. Use of CVD risk score, based on age, total cholesterol, HDL-C, SBP, use of antihypertensive drugs, diabetes, and smoking status, in multivariate models allowed us to account for major CVD confounders without adding many variables that would lead to instability of our models. Of the study limitation, due to potential changes in CVD risk factors during the study follow-up, some degree of misclassification might have occurred which could lead to biased estimated hazard ratios towards the null, as inherent in any prospective study. Furthermore, both the participants’ diets and the composition of food may have changed over the follow-up time, leading to errors in assessing dietary exposure of interest. Moreover, the young age of the participants resulted in low incidence of CVD during the study follow-up.

## 5. Conclusions

In conclusion, our findings provided more evidence to confirm that increased intakes of fiber in a regular diet is an important cardioprotective dietary factor. Both soluble and insoluble dietary fibers have similar beneficial effects against the development of CVD. Dietary fiber intakes from legumes, vegetables and fruits may have stronger impact on cardiovascular outcomes.

## Figures and Tables

**Table 1:** Baseline characteristics of the participants.

	Participants with CVD Outcome (*n* = 57)	Participants without CVD Outcome (*n* = 2202)	*p* Value
Age (years)	58.6 ± 9.7	37.3 ± 13.0	0.001
Male (%)	59.6	42.5	0.001
Smoking (%)	26.3	22.7	0.31
Body mass index (m^2^/kg)	28.4 ± 4.4	26.5 ± 4.8	0.005
Lipid-lowering drugs (%)	10.5	1.5	0.001
Anti-hypertensive drugs (%)	14.3	1.5	0.001
Aspirin (%)	8.9	2.3	0.001
Waist circumference (cm)	97.2 ± 10.1	88.0 ± 13.2	0.001
Serum creatinine (μmol/L)	1.13 ± 0.18	1.03 ± 0.15	0.001
Systolic blood pressure (mmHg)	130 ± 17.4	109 ± 14.9	0.001
Diastolic blood pressure (mmHg)	80.2 ± 10.6	72.5 ± 10.3	0.001
Fasting blood glucose (mg/dL)	106 ± 39.9	88.5 ± 16.7	0.001
Serum triglycerides (mg/dL)	190 ± 107	132 ± 78.0	0.001
HDL-C (mg/dL)	40.2 ± 8.0	43.3 ± 10.4	0.03
TG/HDL-ratio	5.0 ± 3.2	3.4 ± 2.7	0.001
Serum insulin	7.4 ± 3.4	8.8 ± 4.9	0.13
HOMA-IR	1.5 ± 0.8	1.9 ± 0.8	0.28
Hyperinsulinemia (%)	12.5	17.6	0.57
Diabetes (%)	14.3	3.9	0.002
Hypertension (%)	43.9	8.3	0.001
Cardiovascular disease risk score	22.1	21.2	0.001
Dietary intakes			
Total fats (g/day)	74.1 ± 2.5	79.5 ± 0.4	0.03
Saturated fats (g/day)	24.8 ± 1.8	27.1 ± 0.3	0.22
Mono-unsaturated fat (g/day)	21.9 ± 1.0	27.6 ± 0.2	0.01
Sodium (mg/day)	4883 ± 410	4421 ± 66	0.26
Total carbohydrate (g/day)	337 ± 5.8	323 ± 0.9	0.02
Total fiber (g/day)	25.3 ± 1.1	29.0 ± 0.2	0.003

Data are mean ± SD unless stated otherwise (independent *t*-test for continuous variables and chi-square test for dichotomous variables was used.

**Table 2:** The hazard ratio (95% CI) of coronary heart disease across tertiles of dietary fiber and its categories.

Dietary Fiber Intakes	T1	T2	T3	*p* for Trend
Total fiber				
Crude	Ref.	0.83 (0.45–1.54)	0.69 (0.36–1.33)	0.56
Model 1	Ref.	0.87 (0.46–1.62)	0.75 (0.38–1.46)	0.69
Model 2	Ref.	0.67 (0.35–1.26)	0.39 (0.18–0.83)	0.05
Soluble fiber				
Crude	Ref.	0.49 (0.26–0.91)	0.39 (0.20–0.76)	0.01
Model 1	Ref.	0.54 (0.29–1.03)	0.41 (0.20–0.82)	0.04
Model 2 ^a^	Ref.	0.39 (0.21–0.75)	0.19 (0.09–0.41)	0.001
Insoluble fiber				
Crude	Ref.	0.64 (0.34–1.18)	0.53 (0.28–1.02)	0.15
Model 1	Ref.	0.72 (0.39–1.33)	0.58 (0.29–1.14)	0.25
Model 2 ^b^	Ref.	0.54 (0.28–1.03)	0.31 (0.14–0.69)	0.014

Cox proportional hazard regression models were used. Model 1 was adjusted for cardiovascular disease risk score. Model 2 was additionally adjusted for dietary intake of total fats (% of energy), sodium (mg/1000 kcal), and vitamin C (mg/1000 kcal); ^a^ additionally adjusted for insoluble fiber; and ^b^ additionally adjusted for soluble fiber.

**Table 3:** The hazard ratio (95% CI) of coronary heart disease across tertiles of dietary fiber and its categories.

Dietary Fibers	T1	T2	T3	*p* for Trend
Grain fiber				
Crude	Ref.	0.89 (0.46–1.72)	1.11 (0.59–1.07)	0.79
Model 1	Ref.	0.83 (0.42–1.62)	0.98 (0.52–1.84)	0.84
Model 2	Ref.	0.79 (0.39–1.61)	0.90 (0.44–1.86)	0.82
Legume fiber				
Crude	Ref.	0.61 (0.34–1.11)	0.36 (0.18–0.73)	0.01
Model 1	Ref.	0.59 (0.32–1.09)	0.38 (0.18–0.77)	0.02
Model 2	Ref.	0.47 (0.25–0.89)	0.31 (0.15–0.65)	0.003
Nut fiber				
Crude	Ref.	0.78 (0.43–1.41)	0.47 (0.24–0.94)	0.10
Model 1	Ref.	0.77 (0.43–1.42)	0.54 (0.27–1.07)	0.21
Model 2	Ref.	0.65 (0.33–1.27)	0.49 (0.24–1.02)	0.14
Fruit fiber				
Crude	Ref.	0.74 (0.39–1.39)	0.81 (0.43–1.50)	0.61
Model 1	Ref.	0.74 (0.38–1.41)	0.83 (0.44–1.58)	0.65
Model 2	Ref.	0.56 (0.29–1.09)	0.44 (0.22–0.89)	0.05
Vegetable fiber				
Crude	Ref.	0.83 (0.45–1.52)	0.59 (0.31–1.16)	0.32
Model 1	Ref.	0.82 (0.45–1.51)	0.61 (0.31–1.21)	0.37
Model 2	Ref.	0.64 (0.34–1.20)	0.34 (0.16–0.72)	0.02

Cox proportional hazard regression models were used. Model 1 was adjusted for cardiovascular disease risk score. Model 2 was additionally adjusted for dietary intake of total fats (% of energy), sodium (mg/1000 kcal), and vitamin C (mg/1000 kcal), and other types of dietary fiber (g/day).

**Table 4:** The association of dietary fiber intakes and the risk of cardiovascular disease.

Author	Study Population	Findings
Rimm et al. [25]	6-year follow-up study among adult men	Dietary intake of fiber 28.9 vs. 12.4 g/day decreased risk of total myocardial infarction (RR = 0.59, 95% CI = 0.46–0.76) and fatal myocardial infarction RR = 0.45, 95% CI = 0.28–0.72. Cereal fiber reduced risk of total MI (RR = 0.71, 95% CI = 0.55–0.91 for each 10 g/day increase in cereal fiber)
Mozaffarian et al. [26]	8.6-year follow-up of elderly men and women	Highest compared to the lowest quintile of cereal fiber consumption, decreased incident CVD (HR = 0.79; 95% CI = 0.62–0.99) Fruit fiber and vegetable fiber intake were not associated with incident CVD. Higher intake of cereal fiber was associated with lower risk of total stroke, ischemic stroke and ischemic heart disease death.
Streppel et al. [28]	40-year follow-up of adult men	Every additional 10 g/day of dietary fiber intake decreased CVD mortality by 17% (95% CI: 2%, 30%) and all-cause mortality by 9% (0%, 18%).
Threapleton et al. [27]	Meta-analysis of 22 prospective cohorts	Total fiber intake was inversely associated with risk of CVD (RR = 0.91 per 7 g/day, 95% CI = 0.88–0.94) and CVD (RR = 0.91, 95% CI = 0.87–0.94). Each 7 g/day increase in insoluble fiber (RR = 0.82, 95% CI = 0.70–0.96), fiber from cereal (RR = 0.84, 95% CI = 0.76 0.94), and each 4 g/day increase in fiber from vegetable sources (RR = 0.94, 95% CI = 0.89–1.00) decreased risk of CVD and CVD.

MI: Myocardial infarction; CVD: Coronary heart disease; CVD: Cardiovascular disease; HR + Hazard ratio; RR = Relative risk.
